# Out of the bottleneck: the Diversity Outcross and Collaborative Cross mouse populations in behavioral genetics research

**DOI:** 10.1007/s00335-013-9492-9

**Published:** 2013-11-23

**Authors:** Elissa J. Chesler

**Affiliations:** The Jackson Laboratory, 600 Main Street, Bar Harbor, ME 04609 USA

## Abstract

The historical origins of classical laboratory mouse strains have led to a relatively limited range of genetic and phenotypic variation, particularly for the study of behavior. Many recent efforts have resulted in improved diversity and precision of mouse genetic resources for behavioral research, including the Collaborative Cross and Diversity Outcross population. These two populations, derived from an eight way cross of common and wild-derived strains, have high precision and allelic diversity. Behavioral variation in the population is expanded, both qualitatively and quantitatively. Variation that had once been canalized among the various inbred lines has been made amenable to genetic dissection. The genetic attributes of these complementary populations, along with advances in genetic and genomic technologies, makes a systems genetic analyses of behavior more readily tractable, enabling discovery of a greater range of neurobiological phenomena underlying behavioral variation.

## Behavioral implications of the origins of inbred mice

The historical development of the laboratory mouse from the hands of collectors to the laboratory has been particularly consequential to the study of behavior. First noted in early Indian, Chinese, and Japanese historical records, mice were bred, collected and traded for unusual coat appearance and behaviors. These two selection criteria affected mouse behavior in the resulting population for two reasons, (1) the intentional selection for unusual behavioral characteristics such as ‘waltzing’ behaviors, and (2) the ontogenic similarity of the skin and central nervous system resulting in co-selection of abnormal behavioral characteristics with coat color and skin related phenotypes (Wahlsten 1973). As collectors and researchers maintained these mice, further bottlenecking selection occurred. Breeding efforts have long been suggested to select for ease of capture, first in the wild, and later in the cages. In the laboratory, reproductive fecundity may also have been under positive selection. Finally, intrinsic to the inbreeding process is a loss of diversity through stochastic processes and deleterious allele purging in inbreeding depression. In the well documented transition from English mouse collectors through Abbie Lathrop to William Castle and ultimately C.C. Little of The Jackson Laboratory, the population was further detached from its origins in the wild. At Jackson, mice were selectively bred for a host of disease related phenotypes and maintained as inbred strains.

These inbred stocks later became the basis for virtually all experimental intercrosses, heterogeneous stocks, and other research populations that have been used to study genetic variation in behavior, derive selected lines, and develop mutant stocks. The result has been relatively low allelic diversity in the most widely used mouse populations (Roberts et al. 2007), and as a consequence of admixture events, a complex, long-range gametic disequilibrium causing widespread linkage of loci across the genome (Petkov et al. 2005; Payseur and Place 2007). The net effect is to introduce false positive correlations among behavioral phenotypes, where parallel effects of genetic linkage are mistaken for pleiotropic actions of the same polymorphisms. Ultimately, the breeding history has led to limited allelic diversity in particular regions of the genome (Yang et al. 2007, 2011), spurious linkage, and a greatly limited range of behavioral variation relative to wild mice.

## Behavioral genetics in the mouse

The existing mouse population has been productively employed for behavioral genetic analysis for many years (Plomin and Manosevitz 1974; Sprott and Staats 1975; Dewsbury 2009, 2012), and many conventional tests of mouse behavior have been developed to assess behaviors with face or construct validity to psychiatric, pharmacological, and clinical phenomena. Most of these assays are biomedically interesting phenotypes that were originally developed in rats for the testing of pharmaceuticals. They were later extrapolated, often with changes to the apparatus size, to the mouse.

Genetic studies of behavioral diversity reveal heritable variation in neuroanatomical structures, activity, anxiety, novelty seeking, cognition, alcohol and addiction related behaviors, and many others. Although these behaviors have important clinical translational ends, they do not always tap into the natural proclivities of mice. Assays routed in the more ethological characters of nesting, foraging, reproductive behavior, predatory behaviors, escape behaviors, and aggression have been developed and extended for use in the laboratory (Sluyter et al. 1995). A scale for behavioral wildness has also been developed and applied to inbred strains (Wahlsten et al. 2003). These assays may tap into more naturalistic mouse behaviors, and therefore be better indicators of the biological functions underlying cognition, stress, anxiety, reproductive behavior, and cost-benefit decision making as manifested by a rodent. In many cases, application of conventional behavioral assays reveals only a small range of variation among commonly used mouse strains. This constrained variation may facilitate the detection of small genetic effects that are present in the population, a property that has been successfully employed in crosses of closely related strains (Bailey et al. 2008). However, many genes underlying behavior variation are not detectable due to lack of sufficient polymorphism.

## Recombinant inbred panels and the emergence of systems genetics

**Fig. 1:**
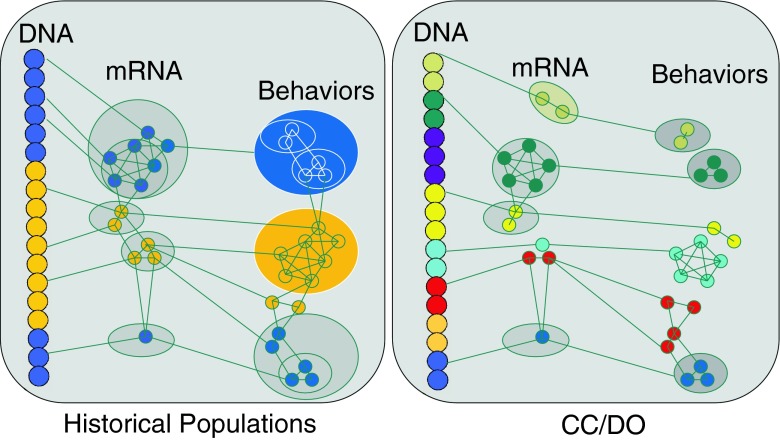
Schematic of the improved precision of systems genetic analysis in historical two progenitor crosses with low recombination density and low precision (*left panel*) versus the Diversity Outcross (J:DO) and Collaborative Cross (*right panel*). More refined recombination structure in the new populations lines result in smaller more refined co-expression networks and correlations among behavioral phenotypes at sample sizes comparable to convention behavioral QTL mapping studies

Isogenic strain panels, notably including recombinant inbred strains, are the key enabling resource for the integration of data across multiple independent measures, realized in behavioral genetics as early as 1977 (Simmel and Eleftheriou 1977). The recombinant inbred strategy was first devised as a technique for constructing linkage maps in immunology, and these were rapidly adopted by behavioral geneticists to go beyond the analysis of heritability of behavior and into the localization of regulatory loci (Crabbe et al. 1980). Behavioral geneticists have long recognized another utility of a genetic mapping panel comprised of isogenic lines-deep replication for precise phenotypic estimation and comparison of independent genome matched controls across conditions (Belknap 1998). Early studies of biobehavioral phenotypes among recombinant inbred strains enabled QTL detection and correlation among behaviors and their underlying neurobiological substrates (Crabbe et al. 1980, 1982; Schoemaker et al. 1982; Goldman and Crabbe 1986) including what may be one of the first ‘systems genetics’ studies of behavior (Goldman and Crabbe 1986) in which localized mouse brain proteins were correlated to phenotypes of alcohol withdrawal and alcohol intake. Systems genetics combine genetic analysis with elements of systems biology including whole system characterization via high-throughput quantitation of biomolecules and data integration across many bio-behavioral measures. In these analyses, a network of relations among the biological entities are constructed through analysis of genetic correlation among these measures. Bulk analysis of gene expression in the brain and behavior became possible with the advent of the microarray, and several systems genetics studies of brain and behavior have been performed in both recombinant inbred and inbred strains (Chesler et al. 2005; Kempermann et al. 2006; Lu et al. 2008; Park et al. 2011; Vanderlinden et al. 2013). Increasingly, researchers have taken advantage of the retrievable nature of recombinant inbred panels, which enable characterization of incompatible measures such as gene expression genetics in naïve and alcohol exposed mice of the same strains (Wolen et al. 2012). The precision of genetic mapping and gene co-expression networks was relatively low in early systems genetics studies (Chesler et al. 2005). The resulting gene-to-behavior networks consisted of large sets of co-expressed genes reflecting multiple diverse biological processes (Fig. 1). However, as with QTL mapping of single traits several systems genetics findings have been resolved to molecular level effects of polymorphisms (Mulligan et al. 2012; Wang et al. 2012). Notable among these are the findings of multiple effects of a common *Comt1* variant (Kember et al. 2010; Li et al. 2010; Segall et al. 2010).

## Improving precision and genetic variation

Many efforts have been made to improve variation and genetic mapping precision in the laboratory mouse since the earliest of QTL mapping studies. Successful genetic analysis of behavior requires high rates of genetic recombination for precise localization of genetic loci, low rates of long-range (i.e., no syntenic) linkage disequilibrium across the genome for accurate localization of genetic loci, and high allelic diversity to maximize the chances of detecting variation among traits. New mouse resources have been continually developed to bring increased allelic diversity, either through a greater number of founders or increased evolutionary distance of founders, and/or increased precision through the assessment of mice with increased numbers of generations, each resulting in additional meiotic recombination events.

The simplest strategy to improve mapping precision is simply to increase the number of recombinations that occur. Advanced intercross lines (AILs) were derived from the continued intercrossing of two founder strains to increase recombination precision (Darvasi and Soller 1995). Typically, an expansion is performed in the last generation of the intercross to create a sufficiently large population for mapping. These populations have been used to map traits with very high precision (Parker et al. 2012a, b), but do require extensive measures to reduce the influence of family structure in the population (Cheng et al. 2010).

An expanded panel of BXD RI lines, made from advanced intercross lines (Peirce et al. 2004) has improved the precision with which traits can be mapped in recombinant inbred lines, and an increasing number of studies report successful identification of QTLs as a result. However, studies in this population remain limited to the detection of effects driven by the polymorphisms between the C57BL/6 and DBA/2 founders. Several widely used behavioral traits surveyed among these strains exhibit insufficient variation relative to within strain variation (Philip et al. 2010). Furthermore, some drift has occurred in the founder stocks between the production of early and late BXD RI lines (Shifman et al. 2006), resulting in allelic effects of opposite direction for at least one of many traits we have examined (Philip et al. 2010).

Historically, several heterogeneous mouse populations were developed to capture the genetic variation of a wide range of laboratory strains for construction of a “selection base” population. The Northport (Hitzemann et al. 1994) and Institute for Behavioral Genetics (Erwin et al. 1980) heterogeneous stocks are two widely used populations characterized for behavioral and longevity related traits. These gave rise to many selected lines in alcohol and addiction research, (Phillips et al. 1989) and a high precision mapping population (Valdar et al. 2006). The LXS recombinant inbred population was developed from inbred alcohol response selected lines derived from this stock (Williams et al. 2004). Mapping methods for the use of widely available commercial outbred mice including the Hsd:ICR stock and many others have recently been developed. These populations have somewhat high genetic diversity depending on colony size, and have extreme recombinational precision (Yalcin et al. 2010; Yalcin and Flint 2012).

Wild derived inbred strains possess many more genetic polymorphisms (Salcedo et al. 2007), rapid decay of linkage disequilibrium (Laurie et al. 2007) for high precision, and tremendous behavioral variation (Fernandes et al. 2004; McShane et al. 2010; Patil et al. 2011). While some constitute separate sub-species, many can be intercrossed with common inbred strains and with each other (Forejt 1985). Wild derived experimental crosses can be used to introduce genetic variation in the vast majority of genes in the genome, increasing the likelihood of detecting a QTL. For example, a PWK/PhJ × C57BL/6J cross was used to map variation in taste preference (Tordoff et al. 2008). Wild derived consomics provide another effort to introduce high genetic diversity into mouse populations (Gregorova et al. 2008). These have been used to study variation and can be a base population for the derivation of consomic lines. Further afield are efforts to genetically characterize behavioral variation in far more distant mouse populations, particularly including *Peromyscus spp*, which was recently characterized for nesting behavior (Weber et al. 2013).

## The Collaborative Cross and Diversity Outcross

Each of the efforts to develop novel mouse populations for genetic mapping of behavior exposes additional allelic variants to investigation, comprising perturbations of biological systems not found in the most widely used inbred mouse strains, and in some cases capturing a range of behavioral variation more reflective of the naturally occurring mouse population. However, each of these populations provides challenges for current deployment in the practicality of maintenance, lack of extant founder stocks for sequence reference and heritability studies, and idiosyncratic mating histories.

In behavioral genetic analyses, the Collaborative Cross (CC) and Diversity Outcross (DO) populations were designed as complementary resources for the precision mapping and correlation of behavioral phenotypes and underlying biomolecular, physiological, and morphological characteristics of the central nervous system and the organismal milieu. Originally, envisioned as a community resource for precision QTL analysis and later as a genetic reference population for multiplicative data aggregation (Threadgill et al. 2002), the CC is a collection of recombinant inbred mouse lines with high diversity, in which a large number of phenotypes across a host of organ systems can be deeply characterized and integrated. Genetic analysis in this population benefits from the potential for replicate sampling within strains, enabling behavioral neuroscientists to study phenotypes in diverse genetic contexts, and to estimate heritable variation in traits that can then be mapped with great precision in the DO. As the number of available completed lines increases, genetic correlation and additional mapping studies can be performed.

The CC (Churchill et al. 2004) was designed by the members of the Complex Trait Consortium to have high allelic diversity, precision, power for genetic mapping, retrievable genome matched mice for replication or trait correlation, and repeatable experiments. In order to obtain all of these properties, initial designs called for only a limited number of recombinant matings. Eight founder strains and a goal of 1000 recombinant inbred lines were proposed. Strains were chosen based on multidimensional scaling of genotypes with some consideration of sensitization for diabetes, cancer, and other phenotypes; and consist of A/J, C57BL/6J, 129S1/SvImJ, NOD/LtJ, NZO/HlLtJ, CAST/EiJ, PWK/PhJ, and WSB/EiJ.

The CC was bred at multiple sites including an early test cross at Oregon Health and Science University (Iancu et al. 2010), Oxford/Tel Aviv University/ILRI (Iraqi et al. 2008), Oak Ridge National Laboratory (Chesler et al. 2008), and Australia (Morahan et al. 2008). Each has now been characterized on a number of physiological and behavioral parameters (Aylor et al. 2011; Durrant et al. 2011; Philip et al. 2011; Thaisz et al. 2012; Ferris et al. 2013). The DO population (Churchill et al. 2012; Svenson et al. 2012) was derived from CC mice at various stages of inbreeding (Svenson et al. 2012), intercrossed in a pseudorandomized fashion indefinitely. The DO founders were chosen from distinct CC funnels. Through an intercross strategy which avoids matings of sibs or first cousins, recombinations are randomized and founder haplotypes are distributed throughout the population. However, in any given sampling of the population, mice with varying degrees of relatedness are present, and therefore population structure is taken into account in mapping analyses (Svenson et al. 2012). The DO population is an extensible population enabling ultra-precise mapping of complex traits through the ability to obtain many mice, enabling high-powered analysis of the many homozygous and heterozygous states. Each individual in the population is unique and additional cohorts can be added to studies over time.

## Behavioral genetics in the CC and DO populations

Our early behavioral studies in the CC breeding population (Philip et al. 2011) and the DO population (Logan et al. 2013) reveal that in many ways, these populations are living up to their promise both in increased genetic precision and in genetic heterogeneity. Characterization of the breeding population of the CC (Chesler et al. 2008; Philip et al. 2011) enabled us to assess the impact of inbreeding and outbreeding on behavioral variation. Several behavioral traits were characterized in the breeding colony, and were part of the first QTL mapping studies in the CC (Aylor et al. 2011; Durrant et al. 2011; Philip et al. 2011). These included hot plate nociception, open field locomotor activity, wildness, anxiety related behavior, and sleep behavior. Other behaviors were recorded, though not expressly quantified; including food grinding and qualitative differences in nesting behaviors obtained through monitoring of sleep and sleep deprivation. Data from early studies in the ORNL CC production colony are now available through the Mouse Phenome Database (http://phenome.jax.org/).

The impact of low behavioral diversity and poorly randomized allele segregation among existing inbred strains is readily apparent in strain surveys of behavior. Surveys of the common inbred strains (Wahlsten et al. 2003) and CC founder strains (Philip et al. 2011) for docility and wildness traits on initial retrieval from the home cage reveal bimodality with little continuity across the distribution of mouse scores. The low range of variation may be due to either many independent genetic loci each of which have been fixed with different docility related alleles in different strain or the strains may have docility related alleles fixed in the same state. In the former case, genetic crosses and transgressive segregation may reveal the underlying genes. In the latter case, the loci cannot be detected because they lack polymorphic variation. Trait co-variation and genetic correlation, the backbone of early behavioral genetics and more recent systems genetics approaches, rely on variation to detect shared biological substrates among phenotypes. Without variation driven by genetic polymorphisms, there is a lack of detectable co-variation. As a result of historical population restriction, the range of behavioral variation and co-variation is restricted in commonly used mouse populations. Similarly, the breadth of behaviors that have been studied is well suited to the conventional laboratory mouse, but does not reflect the natural behavioral range and variation of wild mice.

In almost all the cases, the range of phenotypic variation in the CC and DO matches or exceeds than that of the founder stocks (Philip et al. 2011). However, these studies also revealed that inbreeding and outcrossing depression had some effects on genetic and behavioral diversity, specifically evaluated through wildness scores. Wildness increased as strains were outcrossed, indicating effects of heterosis and multiple allele combinations in driving wildness behavior. Additional outcrossing of the founders reduced this variation, indicating that the high wildness of the wild derived founders was being diluted among the many common and wild derived loci. Importantly, we demonstrated that a trait nearly dichotomous among founders was restored to a range of continuous variation among the CC lines, rendering it amenable to genetic analysis.

These properties indicate the value CC and DO mice as a selection base. The CC and its derivative DO population possess greater allelic diversity than the outbred stocks or existing selection base populations. The response to selection for home cage activity was found to be greater in CC than the Hsd:ICR strain, a conventional outbred stock (Zombeck et al. 2011), indicating both greater diversity and allelic effects segregating in the population.

## QTL mapping in CC and DO mice

QTL mapping in these new populations requires careful modeling of the large number of allelic effects, and consideration of relations among the individuals in the mapping studies. In some cases, existing loci have been replicated, but more often, novel loci, particularly those driven by wild-derived alleles are detected. Perhaps the most striking findings are the high precision with which QTLs for behavioral phenotypes could be mapped. The CC and DO population have been used to map QTL to intervals that are typically less than 5 Mb and in the DO less than 2 Mb with conventional sample sizes of ~300 mice (Philip et al. 2011; Logan et al. 2013). Depending on the nature of founder allelic effects, this can often mean identification of a small handful of candidate loci for effects driven by differences among common alleles. For effects driven by wild derived versus common alleles many more polymorphic genes may be identified. We have observed that the wild-derived alleles often have larger allelic effects than those driven by common alleles (Philip et al. 2011). In several cases, only a single coding gene is found in the QTL interval (Philip et al. 2011; Logan et al. 2013). The availability of sequence data for all of the founder stocks makes it feasible to scan for polymorphisms, structural variants, and non-coding gene regulatory features (Keane et al. 2011; Danecek et al. 2012; Nellaker et al. 2012; Yalcin et al. 2012).

## Anomalous behavior in CC and DO

The most exciting opportunity for behavioral science in the DO population is the expanded repertoire and range of behaviors observed in these mice. It is important to note that behavioral anomaly is not unique to the DO, but may be more prevalent, and in some cases is amenable to direct genetic analysis. The influence of wild-derived allelic variants in the CC and the DO mouse population has led some to question their utility in conventional mouse assays. In our experience, the mice are amenable to conventional assays, but one must carefully evaluate the validity of conventional interpretation. For example, some mice exhibit high exploratory activity, and in many cases, will spend equal amounts of time in both halves of the light-dark box or novelty preference apparatus. Similarly, mice may not habituate to these split-chamber tests of anxiety, risk taking, and novelty seeking over the duration of the test. When a locus is identified that appears to influence outcomes such as percent time in the light, or open field habituation, it is essential to determine whether this is due to a failure to apprehend or attend to the difference between the compartments of the test, or whether it truly reflects the desired measure of avoidance. Therefore, to properly interpret allelic effects on chamber avoidance, one must assess whether there was discrimination between the chambers by mice possessing each allele. Likewise, we assessed whether the difference in rate of change in activity is correlated with overall high activity (Logan et al. 2013). These relations were evaluated by allele, as most mice have the common-derived alleles.

High genetic diversity means that some of the mice are wilder than typically observed in the lab. For technicians inexperienced with mouse handling in general, there may be added stress induced by prolonged capture and restraint procedures. DO mice exhibit high rates of inter-male aggression. This is easily avoided by restricting study to females, or by singly housing males, but neither of these are ideal solutions. Many influences of post-weaning handling, introduction of non-sib cage mates and age at various housing and husbandry events are suspected to influence aggression related behavior. Systematic studies both on a practical level and in the genetics of aggression related behavior are warranted and will likely be quite fruitful.

In contrast to many conventional mouse populations, the extreme range of variation of behavior in the DO may be a substantial source of variation in cage mate behavior. Dominance hierarchies, social behavior and a number of other behaviors may be influenced by the behavior of the other mice in the cage. Tracking and analyzing these effects using social network graph and other analysis strategies may reveal exquisite impacts of naturalistic social and behavioral effects, e.g., enabling modeling of chronic familial stress. Our initial studies of aggressors vs. non-aggressors from separated cages did not reveal behavioral differences. However, further analysis and systematic evaluation are warranted. Often the unusual or prodigious behavior can be mapped to a single wild-derived allele, as we have done with tail-climbing during the tail-suspension assay of depression related behavior (Logan et al. 2013). In summary, the unique behaviors manifested by the DO mice, and the increased prevalence of certain behaviors provide an opportunity for further characterization. Most genetic analysis methods assume a single model will fit all data to predict phenotype from genotypes. Treating unusual DO phenotypes as qualitatively distinct from the primary trait under consideration may require data partitioning, proportional hazards models, and other genetic analysis methods that enable more than one relationship to occur in the data.

## Improved resolution for eQTLs and gene co-expression analysis

Expression QTL analyses in the CC and DO mice promise to alleviate the challenge of large linked co-expression clusters and lack of precision. In the CC and DO, the high allelic diversity presents problems for microarray and conventional RNAseq analyses alike in their bias toward the C57BL/6J genome. The high allelic diversity in the population leads to many cis expression QTLs, and a virtual absence of the trans QTL bands (Ferris et al. 2013). These are now believed to be largely an artifact of linkage disequilibrium and spurious correlation to environmental variables (Kang et al. 2008), but it is possible that their detection in other populations is in part due to highly constrained variation such that less ‘cis’ variation enables detection of more ‘trans’ variation. The DO present a tremendous advance in our ability to discriminate relations among co-expressed genes. By facilitating construction of precise co-expression networks, specific relations from polymorphisms to behavior through biological networks can be identified (Fig. 1). Specific gene co-expression networks, once defined, are amenable to causal manipulation, deep investigation and dissection of the developmental, dynamic and compensatory effects of polymorphic variation. The large contaminated clusters of genetically co-expressed genes emerging from earlier studies (Chesler et al. 2005) rarely could lead directly to such experimentation.

## Expected utility of the CC and DO resources for behavioral science

The expected benefits of the CC and DO population lie in their high recombinational precision, and in the case of the CC population, as an improved genetic reference population in which repeated sampling of the fixed panel of individuals will enable large scale analysis of trait correlations. Studies in BXD recombinant inbred lines reveal the promise of multiplicative data aggregation (Chesler et al. 2003), and to date, remain the largest and most available population for this purpose. Additionally, several thousand phenotypic measures in addition to many gene expression data sets have been obtained in this population; an achievement yet to be obtained for any other population. It will take many years to reach this status in the CC population.

The CC lines, though fewer in number than originally envisioned, have several advantages for behavioral scientists over the existing population of inbred strains. Allelic variants are distributed throughout the panel. Therefore, many mice comprise novel combinations of alleles across loci, increasing the range of observable behavioral variation, previously compressed by selection. Alleles from wild-derived strains are distributed across the panel such that they occur in multiple strains, enabling the variation resulting from these variants to be detected and mapped with precision. The uniformity of genetic relations across the strains reduces bias in genetic correlation and heritability estimates. Furthermore, by sampling mice from multiple strains, one could perform more readily generalizable studies of drug effects, behavioral differences across conditions and all manner of experimental paradigms, rather than constrain one’s research to the often maligned C57BL/6 or outbred stock. The same simple treatment vs. control paradigm is used, but the findings become less limited to a single biological context. As the number of finished CC lines increases, the population is projected to exceed the size of the most existing genetic reference populations. High-density genotypes are available for the CC and ultimately each line may be fully sequenced. Widespread distribution and coherent phenotyping efforts will be required to realize the potential of the CC as a genetic reference population.

Precision in genetic populations depends on the actual size of recombinant haplotype fragments from the founder stocks, and the observed recombination density based on the diversity of founders, population size and the number of generations in which meiotic recombination occurred. Highly outbred stocks contain phenomenal precision and the DO provides both the high sample size and breeding history required for high precision genetics. The precision genetics enabled by the DO population even in the early generations which we profiled can shorten the process of fine-mapping by nearly a decade relative to a conventional F2 population. It is of course necessary to obtain high-density genotypes from each mouse in the population, and it is not possibly to directly correlate measures across distinct individuals. Instead transitive strategies must be developed to relate data through genotypic or phenotypic measures. Although the DO population is not a model of human genetic diversity in that the precise polymorphisms are not necessarily conserved, it possesses naturally occurring perturbation in loci throughout the genome, allowing evaluation of the effects of heterozygosity and polymorphism on a wider range of behaviors and genes than previously accessible. This high diversity will require high sample sizes for many phenotypes, and to fully realize the potential of mapping the additive, dominant, and epistatic effects of many low frequency alleles.

The availability of sequence data from all the founders coupled with dense genotyping and resequencing of the CC lines themselves will enable researchers to make customized behavioral models with particular allelic variants at known loci, and to evaluate systematically the effects of heterozygosity. Through the production of a recombinant inbred cross, commonly referred to as a ‘RIX’ (Tsaih et al. 2005), mice with defined heterozygotes can be bred and characterized by strategic matings of CC mice with known genotypes. Custom RIX mice can be bred to define specific variants to independently validate the effects of loci estimated from DO mapping studies.

## Conclusion

Behavioral scientists have been early adopters and developers of methods and resources in quantitative genetic analysis for the simple reason that the phenotypes under investigation are highly complex under the influence of many genes and many environmental effects. Experimental paradigms and resources that recognize this complexity have been embraced to enable the discovery of novel mechanisms and interventions for disorders of behavior and to understand the relations among the processes of behavioral variation and behavioral change. Multiple advances in genetic strategies, genome and sequence analysis, and the methods for computational integration of experimental data are all required to fully realize the potential of systems genetic analyses of behavior. Improved quantitation of gene expression, metabolites, proteomes, and all the manner of biological entities have significantly improved the facility of complex trait studies in all populations, and have enabled improved prioritization and validation of biological candidate genes and mechanisms. A wealth of complementary resources and gene manipulation technologies expand the arsenal of genetics tools for the study of brain and behavior. The DO and CC represent resources that will match the challenges of behavioral genetics through constrained, yet unprecedented variation in mouse mapping panels, high-precision genetic mapping and network refinement. Distinctive behaviors in these mice present new opportunities to escape the confines of the conventional behavioral tests, unlocking opportunities to find the genetic basis of more naturalistic mouse behaviors, develop new mouse models through selected breeding and discover the relations among behavioral and neurobiological characters.
